# Chronic Oral Epigallocatechin-gallate Alleviates Streptozotocin-induced Diabetic Neuropathic Hyperalgesia in Rat: Involvement of Oxidative Stress 

**Published:** 2012

**Authors:** Tourandokht Baluchnejadmojarad, Mehrdad Roghani

**Affiliations:** a *Department of Physiology, School of Medicine, Tehran University of Medical Sciences, Tehran, Iran. *; b *Neurophysiology Research Center, Shahed University, Tehran, Iran. *

**Keywords:** Epigallocatechin-3-gallate, hyperalgesia, oxidative stress, Diabetic rat

## Abstract

Due to the anti-diabetic and antioxidant activity of green tea epigallocatechin-gallate (EGCG), this research study was conducted to evaluate, for the first time, the efficacy of chronic treatment of EGCG on alleviation of hyperalgesia in streptozotocin-diabetic (STZ-diabetic) rats. Male Wistar rats were divided into control, diabetic, EGCG-treated-control and diabetic and sodium salicylate (SS)-treated control and diabetic groups. For induction of diabetes, STZ was intraperitoneally injected (IP) at a single dose of 60 mg/Kg. EGCG was orally administered daily at doses of 20 and 40 mg/Kg for seven weeks; one week after diabetes induction. Finally, hyperalgesia was assessed using standard formalin, hot tail immersion and paw pressure tests. Meanwhile, markers of oxidative stress in brain were measured. Diabetic rats showed a marked chemical, thermal and paw pressure hyperalgesia, indicating that the development of diabetic neuropathy and EGCG treatment at a dose 40 mg/Kg significantly ameliorated the alteration in hyperalgesia (p < 0.05) in diabetic rats as compared with untreated diabetics. EGCG treatment (40 mg/Kg) also significantly decreased diabetes-induced thiobarbituric acid reactive substances formation (p < 0.05) and nitrite (p < 0.05) content and reversed the reduction of antioxidant defensive enzyme superoxide dismutase (p < 0.05). The results may suggest therapeutic potential of EGCG for the treatment of diabetic hyperalgesia through the attenuation of oxidative stress.

## Introduction

Diabetes mellitus is one of the serious problems worldwide and the number of diabetic people is estimated to be increased markedly by the year 2030 (1). Uncontrolled chronic hyperglycemia in diabetes leads to severe complications including neuropathy, retinopathy, and autonomic dysfunctions. Diabetic neuropathy as observed in some deranged conditions of nociception (*i.e. *hyperalgesia) is the most common complication with an incidence of more than 50% (2). Diabetes-induced deficits in motor and sensory nerve conduction velocities and other manifestations of peripheral diabetic neuropathy have been well correlated with chronic hyperglycemia. Hyperglycemia could also lead to an increased oxidative stress (enhanced free radical formation and/or a defect in antioxidant defenses), advanced glycation end product formation, nerve hypoxia/ischemia, and impaired nerve growth factor support (3- 5). Several studies suggest that an oxidative stress may be one of the major pathways in the development of diabetic neuropathy and the antioxidant therapy can prevent or reverse the hyperglycemia-induced nerve dysfunctions (6, 7). Recent interests are focusing on the use of non-vitamin antioxidants such as flavonoids in reducing the devastating complications of diabetes in experimental animals and patients (8). Plant-based pharmaceuticals including flavonoids have been employed in the management of various mankind diseases (8). They are as an essential part of human diet and are present in plant extracts that have been used for centuries in oriental medicine. Antioxidant properties, reactive oxygen species (ROS) scavenging, and cell function modulation of flavonoids could account for the large part of their pharmacological activity (8-10). Since diabetes mellitus is considered as a free radical mediated disease, there has been renewed interest in the use of flavonoids in diabetes research. Green tea polyphenols are natural plant flavonoids that comprise many types of catechins. Among these, epigallocatechin-3-gallate (EGCG), comprising about one-third of green tea dry mass (11), is a polyphenolic bioflavonoid derived from a variety of plants, especially green tea that is primarily responsible for its beneficial effects. It has been suggested that EGCG could attenuate lipid peroxidation (12), being capable of reducing the risk of type 1 diabetes (13), and exert the hypoglycemic and hypolipidemic effects (14). EGCG has also been proved to exert europrotective/neurorescue activities against oxidative damage and neurodegeneration (15-17). An increasing body of evidence has demonstrated that EGCG possesses neuroprotective effects against a variety of toxic insults and inflammatory neuronal injuries (18-21). Previous studies also showed that green tea extract has neuroprotective effect on the ischemia/reperfusion-induced brain injury through the inhibition of inflammatory stress-induced neuronal cell death (22, 23). Therefore, we designed this study to investigate, for the first time, the effect of chronic EGCG treatment, a free radical scavenger on hyperalgesia in streptozotocin-diabetic (STZ-diabetic) neuropathic rat, using standard formalin and hot tail immersion tests and also to evaluate the role of oxidative stress. 

## Experimental

*Animals*

Male albino Wistar rats (Pasteur’s institute, Tehran, Iran) weighing 225-285 g (10-12 weeks old) were housed in an air-conditioned colony room on a light/dark cycle (21-23°C and a humidity of 30-40%) and supplied with standard pelleted diet and tap water ad libitum. Procedures involving animals and their care were conducted in conformity with the NIH guidelines for the care and use of laboratory animals.

*Experimental procedure*

The rats (n = 56) were randomly allocated and similarly grouped into seven groups: normal saline-treated control, EGCG-treated control at a dose of 40 mg/Kg, saline-treated diabetic, EGCG-treated diabetic groups at doses of 20 and 40 mg/Kg and sodium salicylate (SS)-treated control and diabetic. The rats were rendered diabetic by a single intraperitoneal injection of 60 mg Kg-1 streptozotocin (STZ) (Pharmacia and Upjohn, USA) freshly dissolved in cold normal saline. Control animals received an injection of an equivalent volume of normal saline. One week after the STZ injection, overnight fasting blood samples were collected and serum glucose concentrations were measured using glucose oxidation method (Zistshimi, Tehran). Only those animals with a fasting serum glucose level higher than 250 mg dL-1 were selected as diabetic for the following experiments. The day on which hyperglycemia had been confirmed was designated as day 0. Diabetes was symptomatically verified by the presence of hyperglycemia, polyphagia, polydipsia, polyuria and weight loss in the following weeks. EGCG (Sigma, USA) was administered p.o. at doses of 20 and 40 mg/Kg/day one week after STZ injection for a period of 7 weeks. Dose of EGCG was chosen according to our pilot study and previous studies (24, 25). EGCG was dissolved in normal saline and freshly administered. SS (200 mg/Kg, IP) was administered 1 h before conducting the formalin test as a positive control. Serum glucose level and body weight were monitored at the start and regularly every week until the end of the study. Changes in food consumption and water intake were regularly observed (quantitatively was not measured) during the experimental period. Pain-related behaviors including formalin, hot tail immersion and paw pressure tests were performed at the end of the study as described below.

*Formalin test*

The previously described method was applied (26). Briefly, each animal was acclimatized to the observation box before any testing began. Then, it was given a subcutaneous injection of 50 μL of 2.5% formalin into the plantar surface of one hind paw. It was then immediately placed in a Plexiglas box. Observations were continued for the next 60 min. A nociceptive score was determined for 5-min blocks by measuring the amount of time spent in each of the four behavioral categories: 0, the position and posture of the injected hind paw is indistinguishable from the contralateral paw; 1, the injected paw has little or no weight placed on it; 2, the injected paw is elevated and is not in contact with any surface; 3, the injected paw is licked, bitten, or shaken. Then, a weighted nociceptive score, ranging from 0 to 3 was calculated by multiplying the time spent in each category by the category weight, summing these products and dividing by the total time for each 5 min block of time. The first 10 min post-injection was considered as the early (first) phase and the time interval 15-60 as the late (sec) phase.

*Hot tail immersion test*

Diabetic thermal hyperalgesia was assessed using tail immersion test (27). After the adaptation, rat tail was immersed in warm water (49°C) and the tail flick response latency (withdrawal response of tail) was observed as the end-point response. Each experiment was repeated 4 times for each animal with an interval of 2 min and its average was reported. Meanwhile, a cut-off time of 30 sec was also considered.

*Paw pressure test*

To test the effect of EGCG on mechanical hyperalgesia, the vocalization thresholds of paw pressure in diabetic rats were measured as described by Randall and Selitto (28) using an analgesia-meter. Increasing pressure (32 g sec-1) was applied through a plastic tip onto the dorsal surface between the 3rd and 4th metatarsus of the left hind paw until the rat squeaked. Vocalization thresholds were expressed in grams and the cutoff was 500 g. Threshold measurements were repeated twice and the average was taken.

*Assay of serum MDA and nitrite concentration and erythrocyte SOD activity*

The MDA concentration (thiobarbituric acid reactive substances, TBARS) in the serum was measured as dsecribed before (29). Briefly, 1.0 mL of 20% trichloroacetic acid and 1.0 mL of 1% TBARS reagent were added to 100 μL of serum, then mixed and incubated at boiling water for 80 min. After cooling on ice, samples were centrifuged at 1000×g for 20 min and the absorbance of the supernatant was read at 532 nm. TBARS results were expressed as MDA equivalents using tetraethoxypropane as standard.

Serum nitrite content was assayed by the Griess method (30). Because NO is a compound with a short half-life and is rapidly converted to the stable end products nitrate (NO3) and nitrite (NO2), the principle of the assay is the conversion of nitrate into nitrite by cadmium and followed by color development with Griess reagent (sulfanilamide and *n*-naphthyl ethylenediamine) in acidic medium. The total nitrite was measured by Griess reaction. The absorbance was determined at 540 nm.

For SOD activity, after centrifuging blood samples (3000 rpm, 4°C, 10 min), RBC portion was seperated and made into 5% homogenate using a homogenizer in cold saline solution. Then, after recentrfuging, the supernatant was used for assay. In this respect, a competitive inhibition assay was performed using xanthine/xanthine oxidase reaction-generated superoxide radicals to reduce nitro blue tetrazolium (NBT) quantitatively to blue formazan. Conversion of superoxide radicals to hydrogen peroxide by superoxide dismutase inhibited dye formation and served as a measure of superoxide dismutase activity. Briefly, 0.5 mL of supernatant was incubated with xanthine (50 μmol/L) and xanthine oxidase (2.5 μmol/L) in 50 mmol/L of potassium phosphate buffer (pH = 7.8, 37ºC) for 40 min and NBT was added. Blue formazan was then monitored spectrophotometrically at 550 nm. The amount of protein that inhibited NBT reduction to 50% maximum was defined as 1 nitrite unit (NU) of SOD activity (29). 

*Chemicals*

EGCG was purchased from Sigma Chemical (St Louis, MO, USA) and dissolved in normal saline and freshly administered. Sodium salicylate was obtained from Darupakhsh (Tehran, Iran) and streptozotocin from Pharmacia and Upjohn (New Jersey, USA). All other chemicals were purchased from Merck (Darmstadt, Germany) and Temad (Tehran, Iran). 

*Statistical analysis*

All results are expressed as mean ± SEM. Distribution of our data follows a normal pattern. Significance of difference between two groups was evaluated using unpaired and paired Student’s t-test. For multiple comparisons, one-way analysis of variance (ANOVA) was used. When ANOVA showed significant difference, Tukey’s post-hoc test was applied. Statistical significance was regarded as p < 0.05. 

## Results

*General considerations*

One week after the STZ injection, four rats (one rat from saline-treated diabetic and three rats from EGCG-treated diabetic groups) were excluded from the study due to their serum glucose level lower than 250 mg/dL and/or being dead. 

**Figure 1:**
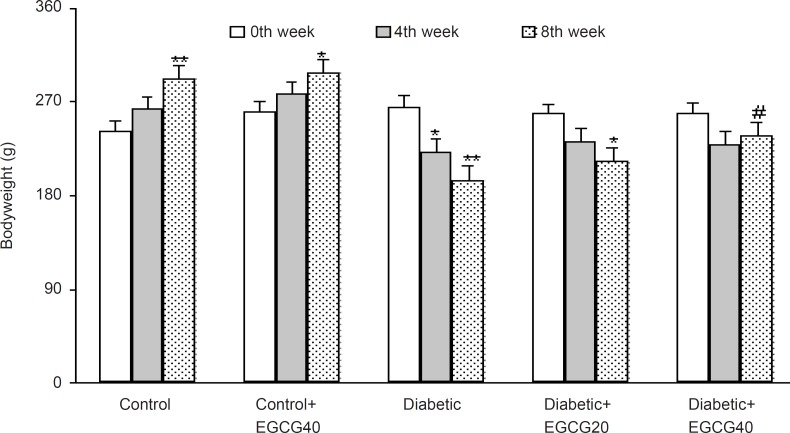
Body weight in different weeks (mean ± SEM). *: p < 0.05, **: p < 0.001 (as compared with week 0 in the same group); #: p < 0.05 (as compared with diabetics in the same week).

**Figure 2:**
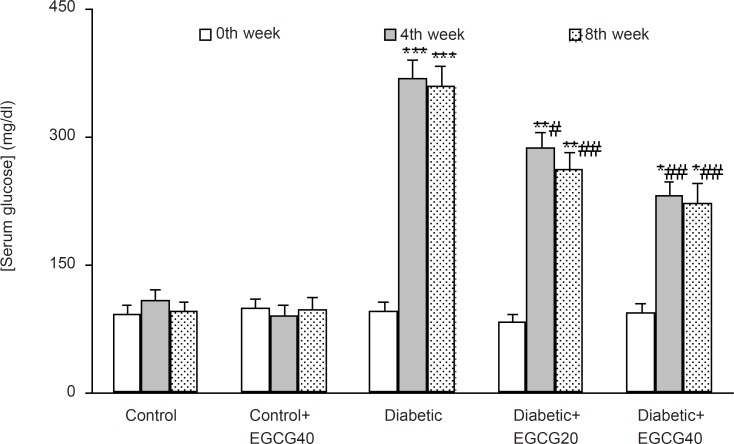
Serum glucose concentration in different weeks (mean ± SEM). *: p < 0.005; **: p < 0.001; ***: p < 0.0001 (as compared with week 0 in the same group); #: p < 0.05; ## p < 0.01 (as compared with diabetics in the same week).

The weight of the vehicle-treated diabetic rats at the 8th week was found to be significantly decreased as compared to control rats (p < 0.01) and EGCG treatment at a dose of 40 mg/Kg caused a less significant decrease in the weight of the diabetic rats as compared with untreated diabetic rats (p < 0.05; Figure 1). In addition, diabetic rats had also an elevated serum glucose level over those of control rats (p < 0.0001) and treatment of diabetic rats with EGCG at both doses of 20 and 40 mg/Kg for 7 weeks caused a significant decrease in the serum glucose (p < 0.01) relative to saline-treated diabetics. Meanwhile, EGCG treatment of control rats did not produce any significant change regarding serum glucose level (Figure 2). 

*Formalin test*

**Figure 3:**
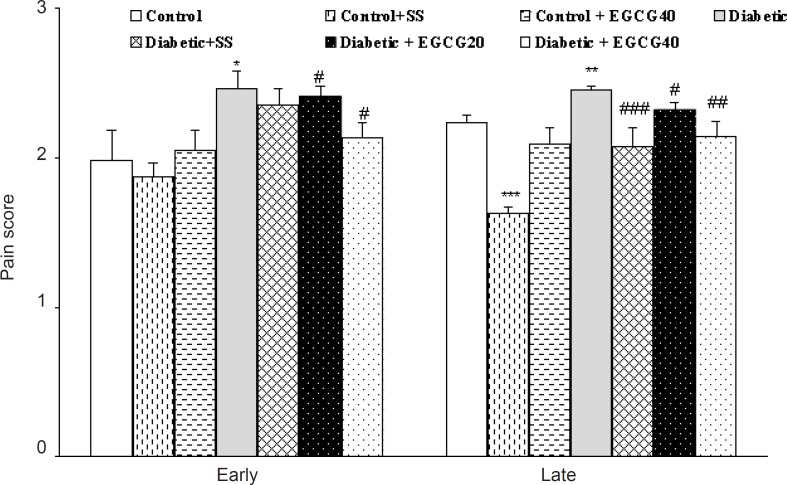
The effect of EGCG and sodium salicylate (SS) on nociceptive scores in the first (early) and second (late) phases of the formalin test. All data represent mean ± SEM. *: p < 0.05, **: p < 0.005, ***: p < 0.0001 (as compared with control); #: p < 0.05, ##: p < 0.01, ###: p < 0.005 (as compared with diabetic).

Hind limb formalin injection produced a marked biphasic response in the rats of all groups. Hyperalgesia was significantly (p < 0.05) greater in untreated diabetics than in control rats in both phases of the test. Pretreatment of control and diabetic rats with sodium salicylate (200 mg/Kg, IP) 1 h before the test caused a significant reduction (p < 0.01) in nociceptive score only in the second phase of the formalin test. In addition, the treatment of diabetic rats with EGCG (40 mg/Kg) caused lower nociceptive scores in both phases of the formalin test as compared to vehicle-treated diabetic rats (p < 0.05). No such response was observed for EGCG (40 mg/Kg)-treated control and EGCG (20 mg/Kg)-treated diabetic rats (Figure 3). 

*Thermal and mechanical hyperalgesia*

**Figure 4:**
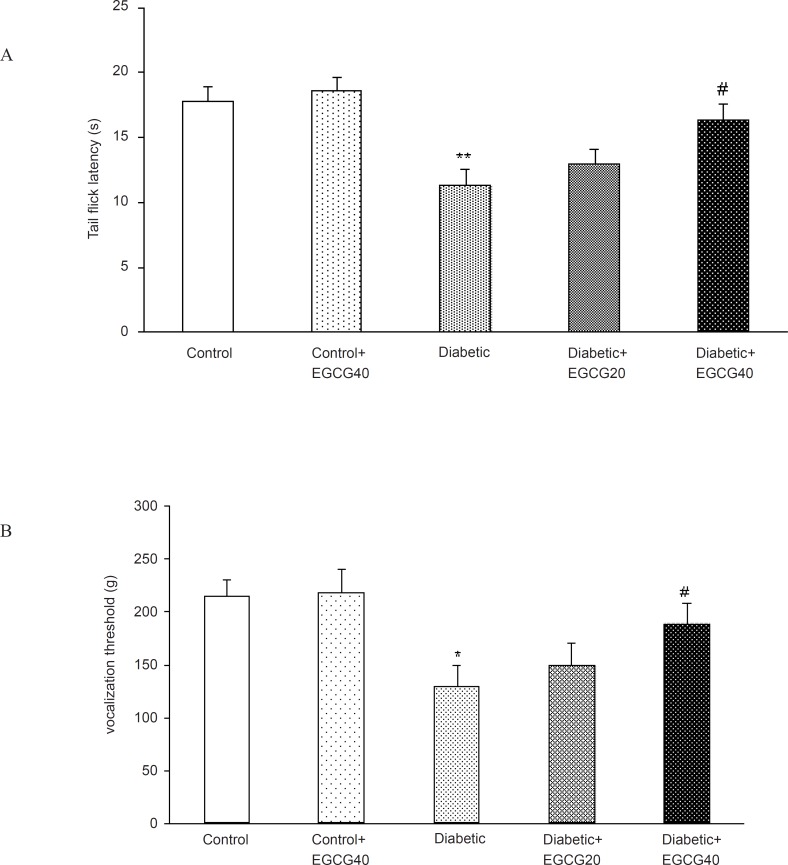
Effect of EGCG treatment on hyperalgesia in hot tail immersion (A) and paw pressure (B) test. All data represent mean ± SEM. *: p < 0.005; **: p < 0.001 (as compared with control); #: p < 0.05 (as compared with diabetic).

A significant decrease in tail flick latency was observed after two months of diabetes in hot tail immersion test (p < 0.01). This deficit in tail flick response latency was significantly (p < 0.05) reversed on treatment with EGCG (40 mg/Kg). To examine the effect of EGCG on mechanical hyperalgesia, diabetic rats were subjected to the Randall-Selitto test. The vocalization threshold in diabetic rats was lower than that in age- matched normal rats (129.1 ± 20.33 g vs. 214 ± 16.2 g, respectively; p < 0.005). EGCG (40 mg/Kg) increased the vocalization threshold in diabetic rats (129.1 ± 20.33 g vs. 188.6 ± 19.3; p < 0.05) (Figures 4).

*Markers of oxidative stress*

**Figure 5:**
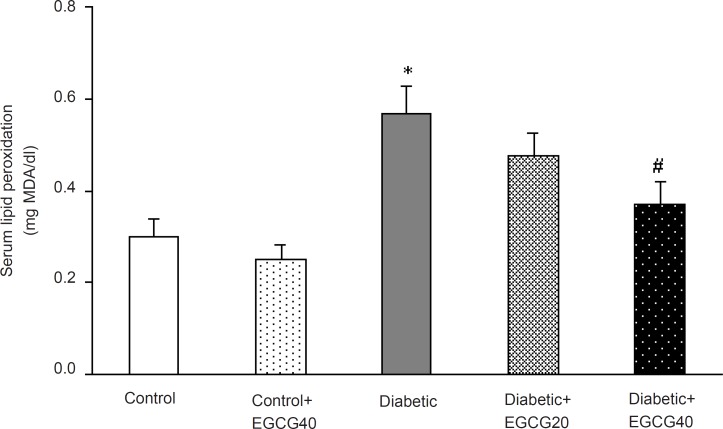
Serum MDA concentration in different groups.*: p < 0.001 (as compared with controls); #: p = 0.02 (as compared with diabetics).

**Figure 6:**
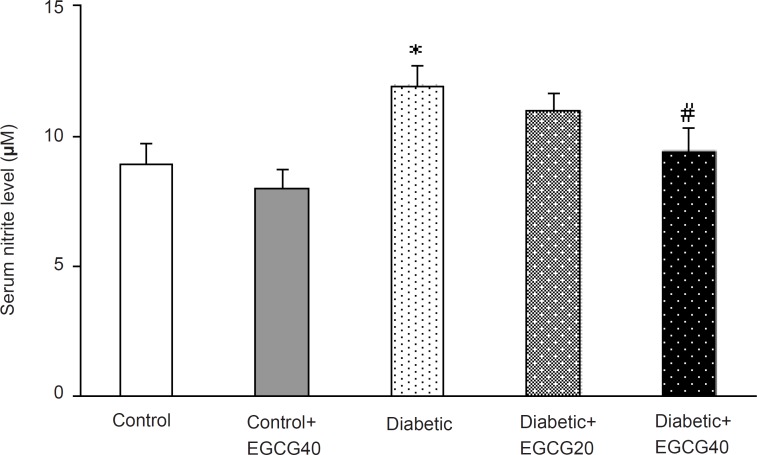
Serum nitrite content in different groups. *: p < 0.01 (as compared with controls); #: p < 0.05 (as compared with diabetics).

**Figure 7:**
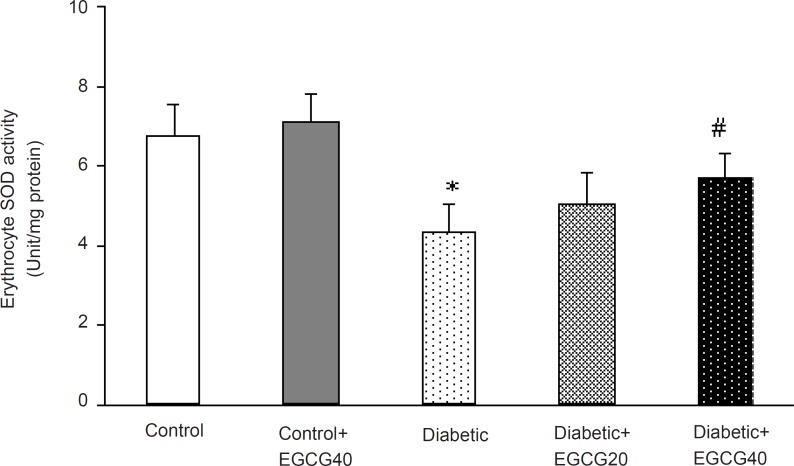
Erythrocyte superoxide dismutase activity in different groups.*: p = 0.03 (as compared with controls); #: p < 0.05 (as compared with diabetics).

Regarding oxidative stress markers, STZ-induced diabetes resulted in an elevation of serum MDA (p < 0.001), nitrite (p < 0.01) content and decreased erythrocyte SOD activity (p = 0.03). Treatment of diabetic group with EGCG (40 mg/ Kg) for 7 weeks significantly lowered MDA (p = 0.02), nitrite (p < 0.05) content and significantly attenuated the reduced activity of SOD (p < 0.05). Meanwhile, there was no significant change in EGCG-treated control group relative to control animals with respect to these parameters (Figure 5-7).

## Discussion

The main findings of this study were three-fold. First, long-term diabetes was accompanied with disturbances in pain-related behaviors, as was evident by a reduction in tail flick latency and vocalization threshold and an intensified nociceptive response in both phases of the formalin test. It is a well-established fact that diabetic rats display exaggerated hyperalgesic behavior in response to noxious stimuli (31) and for this reason STZ-diabetic rats have been increasingly used as a model of painful diabetic neuropathy to assess the efficacy of potential analgesic agents (32). Although the evaluation of mechanisms causing these symptoms is complicated because of the overlap between the systemic effects of hyperglycemia and its toxic effects within the peripheral nervous system, direct functional toxicity of hyperglycemia in the peripheral nervous system (33), an increased activity of primary afferent fibers leading to an increased excitatory tone within the spinal cord, increased release of glutamate and activation of the NMDA receptor, reduced activity of both opioidergic and GABAergic inhibitory systems (34), decreased activity of nNOS-cGMP system in neurons of dorsal root ganglion (35), altered sensitivity of the dopaminergic receptors and altered responsiveness of the dopaminergic system, possibly through the enhancement and/or deactivation of the endogenous Met-enkephalinergic system (36), and alterations in L-type Ca2+ channels (37) could be involved in the modulation of nociception in diabetic rats.

Second, it was demonstrated that oral administration of EGCG at a dose of 40 mg/Kg for a period of 7 weeks could attenuate the reduction in tail flick latency and vocalization threshold and produce a significant analgesic effect in both phases of the formalin test in diabetic rats. On the other hand, sodium salicylate significantly reduced the nociceptive score only in the second phase of the formalin test in control and diabetic rats. It has been known that central acting drugs like narcotics inhibit both phases of the formalin test equally (38), while peripheral acting drugs like aspirin only inhibit the late phase (39). Therefore, the effect of EGCG in this study could be mediated possibly through a central and/or a peripheral mechanism. One of the possible mechanisms that could partially explain the beneficial analgesic property of EGCG may be attributed to its hypoglycemic effect, as observed in this study. In this respect, hypoglycemic effect of EGCG at a dose of 25 mg/Kg for two months has been reported before (14). EGCG has been suggested to inhibit the hepatic gluconeogenesis through a ROS-dependent pathway. EGCG also mimics the cellular effects of insulin such as reducing gene expression of rate-limiting gluconeogenic enzymes (40, 41). Furthermore, EGCG like insulin may increase tyrosine phosphorylation of the insulin receptor and insulin receptor substrate-1 and it reduces phosphoenolpyruvate carboxykinase gene expression in a phosphoinositide 3-kinase-dependent manner. EGCG also mimics insulin by increasing phosphoinositide 3-kinase, mitogen-activated protein kinase, and p70 (s6k) activity (42).

Third, increased free radical mediated-toxicity has also been well documented in clinical diabetes (43) and STZ-diabetic rats (44). Furthermore, oxidative stress is one of the main causes of development of peripheral nerve damage in diabetic neuropathy (45). The elevated level of toxic oxidants in diabetic state may be due to processes such as glucose oxidation and lipid peroxidation. STZ-diabetes is characterized by several derangements in endogenous antioxidant enzymes (43) and the induction of antioxidant enzymes is a critical approach for protecting cells against a variety of endogenous and exogenous toxic compounds such as ROS (44). Numerous studies have shown that green tea flavonoids, have antioxidant and iron chelating activities and thus, can prevent and/or reduce the deleterious effects of oxygen-derived free radicals associated with various chronic diseases (46, 47). Of these, EGCG is the most effective antioxidant (48, 49). Although the mechanisms of EGCG’s antioxidant activity remain unclear, pharmacological studies have identified several antioxidant properties such as: (1) blockade of nNOS and iNOS induction (49-51) and (2) scavenging of free radicals or attenuation of lipid peroxidation (48, 49, 53, 54). Thus, EGCG is expected to be neuroprotective. In our study, chronic EGCG treatment at a dose of 40 mg/Kg for 7 weeks partially reduced lipid peroxidation and nitrite content and improved SOD activity in diabetic rats. Therefore, part of beneficial effect of EGCG in this research could be attributed to the attenuation of oxidative stress in diabetic rats.

To conclude, chronic administration of EGCG could attenuate the hyperalgesic state of diabetic rats and this may be of potential benefit in painful diabetic conditions.
